# Assessment of Diagnostic Skills in Specialist Examinations Should Include Lessons Learnt From Misdiagnosis

**DOI:** 10.1371/journal.pone.0077813

**Published:** 2013-10-16

**Authors:** Ehud Zamir, Kaye Atkinson

**Affiliations:** 1 Centre for Eye Research Australia, University of Melbourne, The Royal Victorian Eye and Ear Hospital, East Melbourne, Victoria, Australia; 2 The Royal Australian College of General Practitioners, East Melbourne, Victoria, Australia; Catholic University of Sacred Heart of Rome, Italy

## Abstract

**Background:**

Poor authenticity in high stake clinical exams adversely effects validity. We propose including known misleading diagnostic factors and contextual biases in the assessment of diagnostic skills amongst advanced specialty trainees. We hypothesise that this strategy offers a more realistic and critical assessment of diagnostic skill than strategies in which candidates are presented with directive, bias free information, allowing for assumptions which cannot be made in real life.

**Methods:**

Eleven patient based practice clinical exam stations were presented to nine advanced ophthalmology trainees. Four patients had a history of misdiagnosis or near misdiagnosis of key ophthalmic findings, presumed to result from identifiable biases and misleading information. In those four stations, candidates were presented with authentic, file based information and were asked authentic questions, similar to those with which the patients presented. If the candidates were unsuccessful in identifying key findings, the questions were converted into directive questions about the same key findings (i.e. “examine the patient’s eyelids, what is your diagnosis?”), and the candidates re-assessed the patient and re-answered.

**Results:**

Ninety-eight doctor-patient encounters took place. Of those, 35 encounters were analysed for the purpose of this study. In 63% of those encounters, key findings were missed when the question included authentic biases or misleading background information, but rephrasing the question to a directive exam format led to their correct identification (Fail converted to pass). Key findings were detected despite contextual biases or misleading background information in only 23% of encounters. In 14% the findings were missed with either question phrasing.

**Conclusions:**

Presentation authentic questions provide a more realistic and less forgiving measure of diagnostic skills than directive exam questions. Given the prevalence of diagnostic errors and their importance to patient outcomes, known mechanisms contributing to diagnostic errors should be used as one of the assessment tools of advanced speciality trainees.

## Background

Speciality training programs use various clinical assessment tools to determine whether the level of competence required of a specialist has been reached by the candidates. Recognition as a specialist is usually the last necessary step in the pathway to independent, unsupervised clinical practice. There is general lack of agreement amongst medical educators about what criteria constitute satisfactory trainee performance in assessments. In this manuscript we will focus on the assessment of diagnostic skills, which constitute an important aspect of clinical competence. We suggest that it is appropriate to use lessons learnt from diagnostic errors as substrate for the assessment of diagnostic skills.

 Misdiagnosis is a realistic, clinically relevant failure of the doctors’ diagnostic skills. It has been estimated to occur at a rate of 10-15% [1,2]. Cognitive errors and biases, rather than knowledge gaps, have been shown to account for the majority of diagnostic errors in clinical practice [2]. Various biases have been described to affect clinicians’ diagnostic performance and underlie the “pathogenesis” of diagnostic errors. [3,4,5,6]

Contextual factors such as low signal to noise ratio in primary care, atypical symptoms, patient’s communication skills and assumptions, incorrect previous diagnoses, and misleading questions posed by previous clinicians are potent diagnostic hurdles in clinical practice. Given that “competence is contextual, reflecting the relationship between a person’s abilities and the tasks he or she is required to perform in a particular situation in the real world” [7], we believe that, to enhance its validity, the assessment of diagnostic skills should include authentic contextual hurdles. 

Current diagnostic skill assessment methods. frequently direct examinees to the area of interest by the questions posed to them or by the title of the examination station (e.g. “Cardiovascular examination”). This by itself introduces a convenient hindsight bias but detracts from the authenticity of the diagnostic task. For instance, in clinical reality there is often uncertainty about the body system/s requiring attention when a patient presents with a symptom. The clinician has to elucidate whether the available information is relevant, the symptom is described accurately, there is a significant finding accounting for the symptom, and there are other important findings the patient is not directing them to. Examinations often present an artificial diagnostic setting in which the answers for the above questions may safely be assumed. For example, a representative question asked in the 2011 Royal Australian and New Zealand College of Ophthalmologists final clinical examination presented a patient with a small medial canthal eyelid tumour. The question started with the direction “examine the medial canthal region of this patient”. In contrast, patients with small canthal skin tumours may in reality have few symptoms and may see the ophthalmologist for an unrelated problem (e.g. diabetic retinal examination). A small, asymptomatic eyelid tumour may easily be missed when it is not the focus of attention, even when the clinician has satisfactory knowledge about lid tumours. All other twenty-two diagnostic questions in that exam followed a similar pattern of directing the candidate to the area of pathology.

A question in the 2011 Royal College of General Practitioners (UK) final exams about a patient with arrhythmia was phrased “A businessman complains of recent intermittent palpitations.” The examiner feedback stated that The purpose of the case is “to test the candidate’s approach to taking a focused cardiovascular history, performing a suitable cardiovascular examination and from this constructing a rational investigation and management plan with the patient. In order to give the candidate time to do a cardiovascular examination if he/she thinks it necessary, much of the history has already been provided.” [8] However, serious cardiac problems may be missed when patients present with less than obvious symptoms (e.g. a brief seizure following hypotension as the presenting symptom of a previously-unknown arrhythmia, rather than straightforward palpitations). Similarly, the Royal College of Physicians (UK) Part 2 Clinical Examination consists of structured system based clinical examination stations, such as respiratory system examination, cardiovascular system stations etc. [9] Furthermore, The MRCPCH clinical examination, held by the Royal College of Paediatrics and Child Health in the UK, are composed of ten stations for each candidate. Examples of stations include: clinical exam: cardiovascular, clinical exam: respiratory, clinical exam: abdominal, clinical exam: Neurological/neurodisability etc. [10]

The above example questions and examination settings might allow adequate assessment of knowledge about lid tumours, cardiac dysrhythmias or respiratory findings. However they may not capture the skill of finding an asymptomatic eyelid tumour in a patient presenting for routine follow up of glaucoma, nor the patient with an atypical, non-cardiac symptom resulting from arrhythmia, or who may have previously been mislabelled as having a gastrointestinal problem when in fact their problem is cardiologic. These hypothetical diagnostic hurdles are real and are routinely present in clinical practice. Doctors who may have adequate knowledge about each of those diseases may still fail to think about them or detect them in the setting of contextual and other biases in real life practice. Therefore, we suggest that the ability to “jump over diagnostic hurdles” is a relevant measure of clinical competency which needs to be assessed in experienced, high level speciality trainees, amongst other measures. 

The study of diagnostic errors may identify authentic, realistic and clinically-proven biases and provide valuable insight into common diagnostic pitfalls. By using such insights, assessments may better measure doctors’ ability to cope with bias. In this way, exams may gain more relevance to clinical outcomes and patient safety. 

We therefore hypothesised that authentically biased clinical scenarios are less likely to be correctly diagnosed by trainees than unbiased presentations of the same clinical problems. We postulate that the use of directive, unbiased exam questions in high stake assessments leads to overestimation of doctors’ diagnostic performance.

We tested our hypothesis in an observational study which will be presented below. 

## Ethics Statement

The project was assessed and approved by the Human Research and Ethics Committee at the Royal Victorian Eye and Ear Hospital. As the practice examination was part of the hospital’s exam training routine and results were unidentified, it was determined by the Human Research and Ethics Committee that verbal consent was sufficient. All participants provided verbal consent, which was documented in the medical files and study files. Patients were informed that their attendance was completely voluntary and was intended for training purposes only. They were given an explanation about the nature of the practice exam and told they would be examined by several doctors multiple times. They all consented to participate. Trainees in various stages of preparation for their final specialty examinations were invited to participate. They were advised that there was no obligation to participate and there would be no consequences for non-participation. They were informed that no identifiable data about their performance in the practice exam was to be collected. They all consented to participate.

## Methods

We conducted an experimental practice clinical exam which was voluntarily taken by 9 ophthalmology registrars. The exam was composed of eleven stations, each simulating an outpatient clinic encounter with a real patient, in a modified objective structured clinical examination (OSCE) format. Eleven patients with significant clinical and/or or ancillary test findings were selected from a general ophthalmology clinic and gave consent to participate. Four of the patients were selected on the basis of previous diagnostic errors. Based on a classification used by the Australian Patient Safety Foundation, we defined diagnostic error operationally as a diagnosis that was unintentionally delayed (sufficient information was available earlier), wrong (another diagnosis was made before the correct one), or missed (no diagnosis was ever made), as judged from the eventual appreciation of more definitive information.

The four study patients were selected because they had important key findings which had been missed or nearly missed by specialist ophthalmologists in the past, without adverse consequences. Key findings were defined as diagnostic physical findings of therapeutic or prognostic significance. In each case, after the correct diagnosis had been reached, the factors contributing to misdiagnosis were studied in hindsight. In each case, one or more biases and misleading factors were identified as the presumed reason for misdiagnosis or near misdiagnosis, and were reproduced for the purpose of this study. The four patients are referred to as misdiagnosis patients (MP). The clinical information about the four MP cases is presented in Table 1. The remaining seven patients had straightforward presentations (SP), with findings directly related to the symptoms and to the available information, and without identifiable biases or misleading data. 

**Table 1:** Clinical features, diagnostic biases and questions asked in the four misdiagnosis patients.

Patient #	Key findings and biasing factor	Original (biased) question phrasing*	New (“standard”) phrasing
1	The patient presented for a routine follow up after an episode of episcleritis which have resolved with topical anti-inflammatory treatment. There were significant but subtle incidental findings: Slight eyelid skin hyperpigmentation on one side and heterochromia (darker iris and sclera in the same eye), indicating oculodermal melanocytosis, a condition which predisposes to ocular melanoma and glaucoma and requires lifelong follow up. Biasing factors: the bias is introduced by the context and the previous diagnosis, which was made by another clinician who had missed the key findings. We refer to this mechanism as “inheritance bias”	This patient is referred due to ocular irritation and itch. What are your findings and recommendations?	Examine this patient’s irides. What are your findings and what is their significance?
2	The patient was under long term follow up for open angle glaucoma and was referred by an optometrist for ongoing care, questioning the adequacy of her glaucoma control. Incidentally, she had several peripheral choroidal nevi, which require lifelong follow up as they may undergo malignant transformation. Biasing factors: a previous diagnosis (“inheritance) and “satisfaction of search” by the obvious glaucomatous findings in the optic nerve head, stopping the examiner from scrutinising the peripheral fundus.	This patient has had open angle glaucoma for several years. Attached are visual field test results and data about the past intraocular pressures and current treatment. The referrer wishes to know whether you think glaucoma control is sufficient.	Examine this patient’s ocular fundi. What are your findings and what is their significance?
3	The patient was under long term follow up for open angle glaucoma and was referred by another ophthalmologist for ongoing glaucoma care. He was known to have “glaucomatous” visual field defects. Careful examination of his computerised visual fields revealed bilateral, small homonymous paracentral defects, indicating a neurological problem. An occipital stroke was revealed by a CT scanBiasing factors: Previous “labelling” as glaucoma (inheritance bias), satisfaction of search: obvious glaucomatous findings in the optic nerve head lead to premature closure and failure to search for alternative explanations for the visual field abnormality.	This patient is referred for ongoing glaucoma management. There are known glaucomatous visual field defects, see attached.	Examine this patient’s visual fields. What are your findings and what is their significance?
4	The patient was referred by another ophthalmologist for ongoing monitoring of a mild, stable macular degeneration in his only eye. As an incidental finding, subtle signs of ocular cicatricial pemphygoid were then detected, The condition is vision threatening if allowed to progress and requires immunosuppressive therapy to control. Biasing factors: Previous information (inheritance bias) and search satisfaction by “zooming in” on the obvious macular lesions and neglecting to see the subtle signs in the conjunctiva.	This patient is referred for ongoing monitoring of his dry age related macular degeneration. There are known glaucomatous visual field defects, see attached.	Examine this patient’s ocular surface. What are your findings and what is their significance?

* visual acuity, intraocular pressures, current medications (ocular and systemic when applicable) were provided for each patient

Trainees were advised that the patient information provided in the practice exam was authentic and unprocessed. Rather than answer traditional exam questions, they were instructed to provide their conclusions as they would do under similar circumstances in their daily clinical routine. SP and MP stations were arranged at a random order. Each patient was presented to the trainees in a short written summary which included all the historical and clinical information available at their original presentation, including ancillary test results. Open clinical questions were asked (e.g. “what are your findings and recommendations?”) while directive questions (e.g. “examine the patient’s ocular fundus, what are the findings? How would you treat them?”) were avoided. Candidates were then allowed 8 minutes to examine the patient and provide their opinion. They were free to interact with the patient as they would in a normal consultation. In each MP case, the candidate’s response was then noted with regards to identification of the key clinical and/or ancillary test findings. If the key findings were missed by the candidate, the station supervisor re-phrased the question and posed it in a directive, “classical” exam format (e.g. examine this patient’s fundi/ anterior segment/ eyelids etc). The candidate then re-examined the patient and their final response to the question was noted. The questions asked about each of the four MP patients are presented in Table 1.

## Results

The results are summarised in Table 2. Data were available for 35 MP patient doctor encounters (one station was accidentally missed by one of the trainees). 

**Table 2:** Trainee performance in the four “misdiagnosis” stations.

Trainee #	Patient 1	Patient 2	Patient 3	Patient 4
1	- +	- +	- -	- +
2	- +	- +	- -	- +
3	- +	- +	- -	- +
4	- +		- +	+
5	+	+	+	- -
6	- +	- +	+ -	- +
7	- +	- +	- +	+
8	+	- +	- +	- +
9	+	- +	- -	- +

+ trainee identified the key findings at first attempt, when presented with authentically-biased information. - + trainee missed the key findings at first attempt, when presented with authentically-biased information, but correctly identified them when presented with a directive, unbiased question.

- - trainee missed the key findings with either phrasing

Only in 8 out of 35 encounters (23%), trainees correctly identified the key findings when presented with authentic (biasing) information. In contrast, the key findings were correctly identified in 29 out of 35 encounters (82%) when presented with directive questions. In 22 encounters (63%), trainees missed the key findings when presented with authentic biases or misleading background information, but answered correctly after questions were re-phrased and bias removed. In the remaining 5 encounters (14%) the key findings were missed with either question method. Of the 27 encounters where the diagnoses were missed with authentic questioning, 22 (81%) converted from “fail” to “pass” after rephrasing of the question. 

in all cases of misdiagnosis in the exam, the candidates followed the pathway of bias that we had predicted from the patient’s history of misdiagnosis, and were misled by the presentation or by inaccurate previous diagnoses:

Patient 1: in all six misdiagnosis encountered the candidates referred to items relevant to the misleading information, namely ocular surface findings. They therefore failed to note the heterochromia. When prompted to look at the patient’s irides, all six candidates noticed the heterochromia and the features of oculodermal melanocytosis and corrected their diagnosis.

Patient 2: in all seven misdiagnosis encountered the candidates examined the relevant items to glaucoma assessment. Candidates only examined the anterior segment and only included the optic nerve head in their fundus examination. None of the candidates examined the more peripheral fundus. When prompted to look at the ocular fundus, all seven candidates noticed the choroidal nevi and corrected their diagnosis.

Patient 3: in all seven misdiagnosis encountered the candidates examined the relevant items to glaucoma (anterior segment anatomy, optic nerve head morphology, and an assessment of the computerised visual field results, examining one-eye visual field printout at a time. They all interpreted the visual field defect as consistent with glaucoma. When prompted to only examine the visual field, three candidates noticed the homonymous defect and corrected their diagnosis.

Patient 4: in all misdiagnosis encountered the candidates referred to items relevant to the information provided (macular degeneration). They examined the ocular fundus while overlooking the subtle ocular surface findings. When prompted to examine the ocular surface, six candidates noticed the pathological signs of ocular cicatricial pemphygoid and corrected their diagnosis.

Interestingly, trainees showed consistency in their pattern of diagnostic skill, with 7 out of 9 trainees (1–3,5–8) following the same answer pattern in 3 of the 4 stations (75%) for each trainee. 

## Discussion

When assessing the clinical competence of speciality trainees to practice as specialists, the skill of crossing diagnostic obstacles needs to be considered. It has been shown by Graber et al [2] that when specialists misdiagnose serious medical conditions they rarely do so because of lack of detailed knowledge about them. They are more likely to miss a diagnosis because of not thinking about it, or being misled towards a different diagnostic direction by biasing factors and faulty information processing. There is a debate about the utility of bias awareness in improving the outcome of the diagnostic process and reducing the likelihood of misdiagnosis [4,11,12]. Without taking a stand on the trainability of doctors in that regard, we suggest that this skill is assessable, similar to the skill of performing an operation, managing surgical complications or reading an ECG. 

Although our sample is small, one of the trainees in our series (number 5) consistently identified the key clinical findings in 3 out of 4 stations despite the biasing presentation. This was in contrast to other trainees who had the inverse ratio of correct diagnosis. It is conceivable that the ability to “see through” misleading information is a skill, rather than a random event. However one of the limitations of our study is the small number of stations with diagnostic “hurdles”, and we suggest that a larger sample size per trainee will be required to reliably identify trends of weakness or strength if this principle is adopted into routine assessments. The design of our experiment allowed candidates to re-assess the patient after forming their initial impression. Before reassessing, they were given directive questions, leading them to the findings they had previously missed. It is possible that in some cases, the opportunity to reassess the patient by itself helped in reaching the correct diagnosis, rather than the switch from an authentic question to a directive one. In that case, our observed increase in the diagnostic accuracy (81%) may be an exaggerated estimate. Using similar methodology, Arzy et al [13] found that asking attending doctors to formulate an alternative diagnosis to their original one after omission of a misleading detail, the diagnostic error rate was reduced by nearly 50%. 

Diagnostic skills need to be identified by a valid, authentic challenge, as a legitimate part of our assessment of specialty trainees. Failing to assess these skills in an authentic manner defeats the very purpose of patient focused high level specialty trainee assessments. 

In 77% of the clinical encounters with the more diagnostically challenging patients in our study, the candidates misdiagnosed the problem when presented with authentic misleading factors. However, in 82% of those misdiagnosed cases, candidates would still pass the exam when presented with processed, bias free information and asked directive questions. Our results therefore indicate that the elimination of common authentic biases from clinical exam questions results in unrealistically robust exam performance, and may overestimate the candidate’s ability to correctly diagnose a similar patient in real life. 

We used a specialist ophthalmology setting for our study. We believe the principles shown by our results are applicable to diagnostic situations in various specialties, including those providing primary care (e.g. general practice, emergency medicine, general medicine, paediatrics). Most commonly, the bias detected in our patients’ cases and reproduced in the study included the presence of an incorrect previous diagnosis, which we call “inheritance bias”. It has been shown by McLaughlin et al [14] that suggesting an incorrect initial diagnostic hypothesis to emergency physicians adversely affects their information processing and leads to poor diagnostic performance.

One of the key problems contributing to misdiagnosis in the primary care setting is the difficulty in detecting the infrequent, serious finding (e.g. a brain tumour), amidst a background of the more frequent, low level and less significant clinical presentations (e.g. tension headaches). This “low signal to noise ratio” setting requires particularly robust diagnostic skills (5).

**Figure 1:**
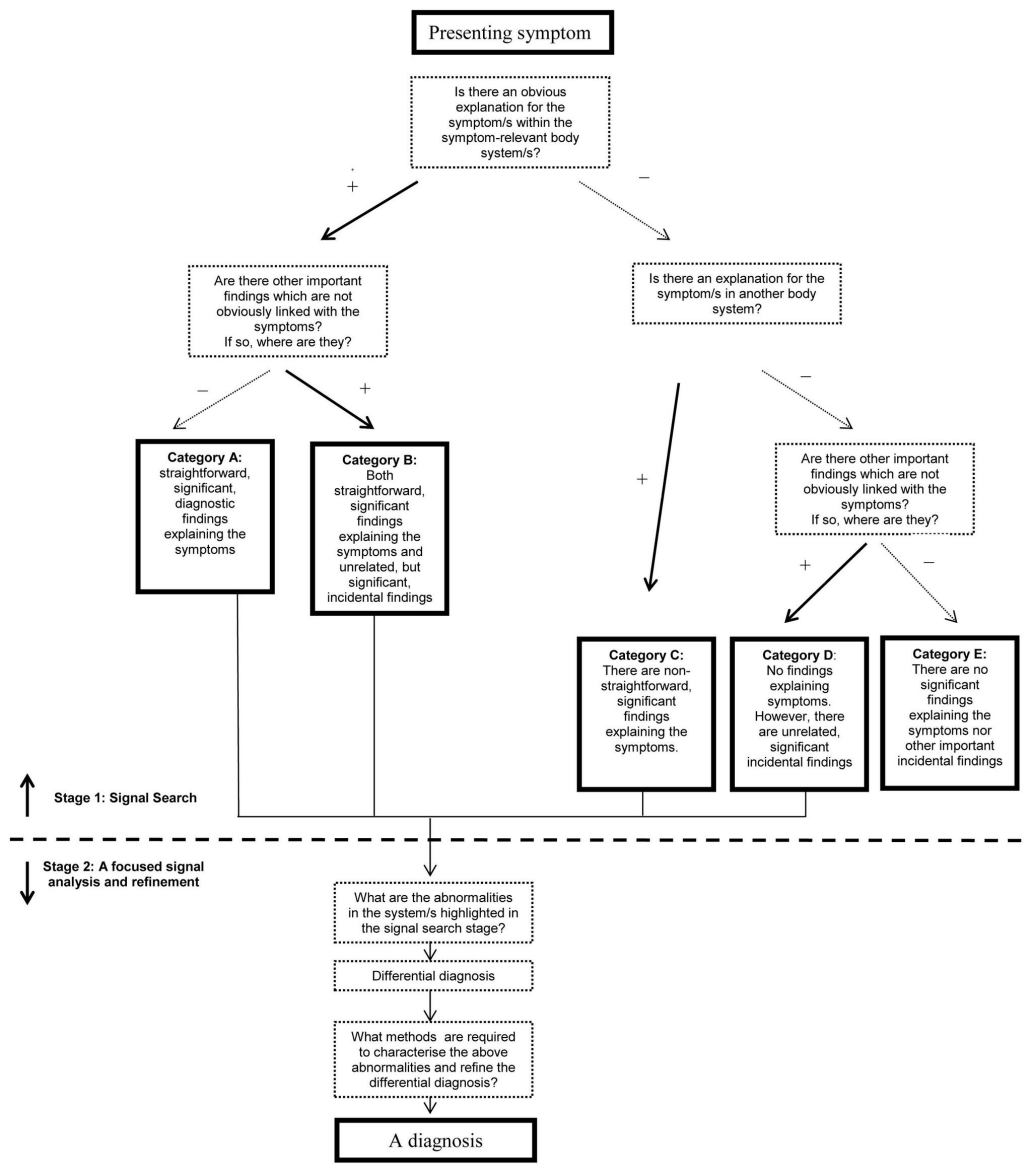
A Theoretical diagnostic algorithm in real life.

We would like to propose a schematic algorithm for the cognitive steps and questions a physician has to consider during the diagnostic process in this setting. A graphical summary of this proposed algorithm is depicted in Figure 1. We divide the cognitive diagnostic process into 2 main stages. 

### Stage 1

Signal search. The aim of this stage is to determine whether the case is a “signal case” and the patient has a significant finding, and in what organ system the finding is. To establish that, the following questions are considered:

• Is there an obvious explanation for the symptom/s within the symptom relevant body system/s?
• Is there an explanation for the symptom/s in another body system?
• Are there other important findings which are not obviously linked with the symptoms? If so, where are they?

These questions must be addressed and answered correctly by the doctor before a more focused differential diagnosis is formed in stage 2. The output of this stage is usually one of five presentation based diagnostic categories, A to E, as shown in Figure 1. As an example, a patient who complains of left shoulder pain may be found to have supraspinatus tendinitis (category A). There may be an additional finding of a suspicious pigmented skin lesion on his back (category B). The patient may have no shoulder findings, but a suspicion may arise that his shoulder pain represents cardiac ischaemia (category C). There could be no significant findings to account for his shoulder pain, but an incidental finding of anaemia (category D). Lastly, there may be no significant findings to be found at all (category E). In our experiment, all our MP patients may be classified as category B by this classification, having both an obvious finding accounting for their presentation and a significant but unrelated finding elsewhere. In our MP cases, the diagnostic errors involved misclassifying the patients as category A rather than B in stage 1. 

### Stage 2

Focused analysis and refinement of the signal found in stage 1, to determine the significance of the signal and reach a final diagnosis.

• What are the abnormalities in the system/s highlighted during the signal search stage?
• What methods (diagnostic tests, further consultation etc) are required to characterise the above abnormalities and refine the differential diagnosis?
• What is/are the diagnosis/es?

This division is a schematic, simplified model of the diagnostic process, in which the two stages do not necessarily occur sequentially. In many cases parts of the two stages overlap in time, according to the individual doctor’s diagnostic style and the case circumstances.

**Figure 2:**
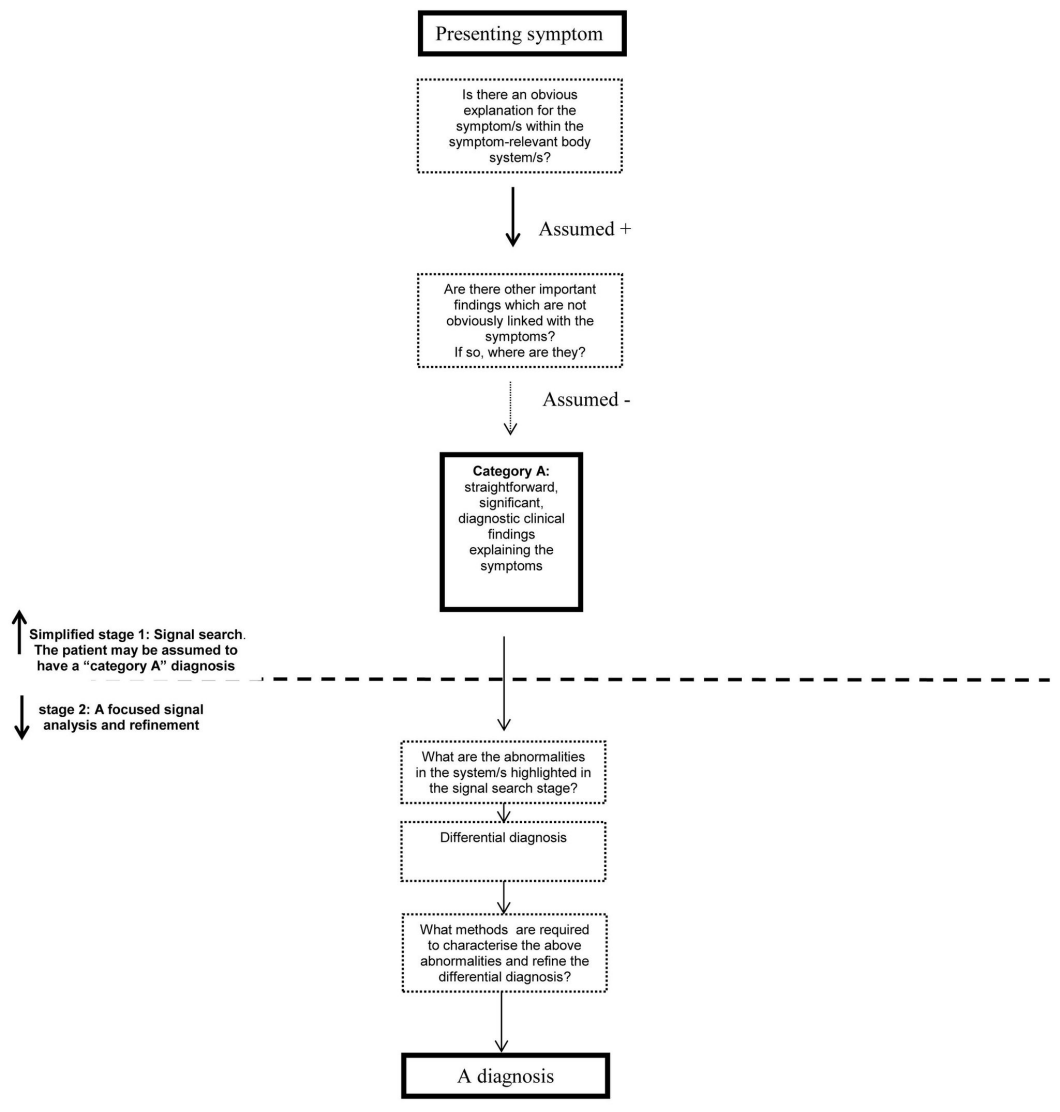
A theoretical diagnostic algorithm in typical, directive clinical exams.

We suggest that exam questions should attempt to assess stage 1 performance and not allow for assumptions about the answers to stage 1 questions to be made (Figure 2). That is, patients should not be assumed to have a “category A” diagnostic situation, with a significant, straightforward diagnosis which is relevant to the presenting symptom/s or exam question. In clinical practice such assumptions cannot be made, and failing to correctly perform “stage 1” may lead to serious misdiagnosis or to unnecessary testing (in the case of “category E” patients, Figure 1). For example, fatigue is a nonspecific, common complaint in general practice. Detecting splenomegaly in a one out of 100 fatigued patients indicates sound “stage 1” skills. Once splenomegaly is identified, then performing a knowledgeable and competent work up for this finding and reaching a diagnosis of myelodysplastic syndrome indicates sound “stage 2” skills. Asking an exam candidate to examine the abdomen of a fatigued patient in an exam skips stage 1 as it implies that there is an abdominal finding related to the symptom. We postulate that it is more likely for a primary care physician to miss splenomegaly in a fatigued patient, than to fail to conduct appropriate investigations leading to a correct diagnosis once splenomegaly is detected. 

Assessment of “stage 2” diagnostic skills and knowledge is usually the focus of clinical examinations. Yet, “stage 1” is a critical part of the diagnostic process. Regardless of specialty, advanced trainees should be assessed on their skill to overcome common diagnostic hurdles. 

We believe that our model of diagnostic skill assessment has high face validity. It uses real patients and allows reproduction of diagnostic errors which have occurred to real patients in the hands of real doctors. This model may also be used as a clinical diagnosis teaching tool. Logistically, this model is labour intensive. It requires identifying patients who have been misdiagnosed, analysing and reproducing the misdiagnosis in a controlled environment. However, questions about patients with authentic diagnostic “twists’ should constitute an unpredictable minority of the overall exam questions (as is the case in real life: most cases are straightforward). The pass rate in authentic questions will probably be lower than in directive questions. Consistent success or failure in this type of assessment should be used as an important indicator of diagnostic skills. It may be that the optimal assessment format which will lend itself to our proposed method will be work-based clinical simulations with standardised patients. This method is considered the most accurate way of assessing clinician’s behaviour [7]. However, we have shown that using our method in an OSCE format is feasible and provides valuable, clinically-relevant information. 

In conclusion, we have demonstrated that lessons learnt from misdiagnosis may be used to improve the authenticity and accuracy of diagnostic skill assessments. Because the skill of overcoming diagnostic obstacles is a key component of independent specialist practice, we believe it should be assessed at high stake specialty exams. 
